# A review of skin microbiome and new challenges to cosmetic microbiome‐friendly formulations

**DOI:** 10.1111/ics.70073

**Published:** 2026-01-12

**Authors:** Yasmin Rosa Santos, Newton Andréo‐Filho, Patricia Santos Lopes, Vânia Rodrigues Leite‐Silva

**Affiliations:** ^1^ Programa de Pós‐Graduação em Medicina Translacional, Departamento de Medicina, Escola Paulista de Medicina Universidade Federal de São Paulo Diadema Brazil; ^2^ Departamento de Ciências Farmacêuticas, Instituto de Ciências Ambientais, Químicas e Farmacêuticas Universidade Federal de São Paulo, UNIFESP – Diadema Diadema Brazil; ^3^ Therapeutics Research Centre The University of Queensland Diamantina Institute, Translational Research Institute Brisbane Queensland Australia

**Keywords:** cosmetics, fragrances, preservatives, skin microbiome, UV filters

## Abstract

Human skin is a complex ecosystem that hosts diverse species of microorganisms. Unbalanced conditions caused by intrinsic and/or extrinsic factors can lead to dysbiosis, presenting symptoms, such as dryness, high transepidermal water loss, reduced barrier protection, premature ageing, and in severe cases, inflammatory dermatoses. Strategies to maintain the skin microbiome balance are becoming increasingly suggested, with prebiotic, probiotic, or postbiotic ingredients promoting the diversity and relative abundance of important microorganisms. Topical products directly influence this balance, both traditional ingredients and specific active ingredients. The concentration and combination of these ingredients, as well as the pH of the final product, are extrinsic characteristics that can affect homeostatic skin condition. Focused on repairing or preserving the skin microbiota, microbiome‐friendly cosmetics are gaining prominence in the cosmetics industry, with a focus on reducing or replacing ingredients with adverse effects on skin microbiota or adding positive compounds for the microbiota. This review approaches the main characteristics of the skin microbiome, in symbiosis and dysbiosis, elucidates strategies for skin microbiota rebalance, and addresses the challenges of developing microbiome‐friendly products through studies of the interaction between skin microbiome and substantial classes of cosmetic ingredients, such as surfactants, lipophilic compounds, preservatives, fragrances, vitamins, and UV filters. The presented findings elucidate the relationship between the host, the skin microbiome, and the use of cosmetics, which could serve as a tool for the development of microbiome‐friendly cosmetics. Given the growing popularity of this topic, we also highlight the need for further research focused on the dynamics between the skin microbiome and cosmetic ingredients.

## Graphical abstract

Microbiome‐friendly concepts offer new perspectives for a healthy relationship between cosmetic use and the skin microbiome, especially the interaction of non‐biotic cosmetic ingredients with the skin, requiring further studies to clarify the dynamics between the skin microbiome and different classes of cosmetic ingredients.

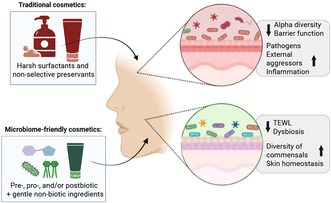

## RÉSUMÉ

La peau humaine est un écosystème complexe qui héberge diverses espèces de micro‐organismes. Des conditions déséquilibrées causées par des facteurs intrinsèques et/ou extrinsèques peuvent entraîner une dysbiose, l’apparition de symptômes, tels qu’une sécheresse, une perte d’eau transépidermique élevée, une protection barrière réduite, un vieillissement prématuré et, dans les cas sévères, des dermatoses inflammatoires. Il est de plus en plus suggéré d’adopter des stratégies visant à maintenir l’équilibre du microbiome cutané, avec des ingrédients prébiotiques, probiotiques ou postbiotiques favorisant la diversité et l’abondance relative de micro‐organismes importants. Les produits topiques, qu’ils soient à base d’ingrédients traditionnels ou de principes actifs spécifiques, influencent directement cet équilibre. La concentration et la combinaison de ces ingrédients, ainsi que le pH du produit final, sont des caractéristiques extrinsèques pouvant affecter l’état homéostatique de la peau. Centrés sur la réparation ou la préservation du microbiote cutané, les cosmétiques respectueux du microbiome prennent de l’importance dans l’industrie cosmétique, en se concentrant sur la réduction ou le remplacement des ingrédients ayant des effets indésirables sur le microbiote cutané ou sur l’ajout de composés ayant un effet positif sur le microbiote. Cette revue aborde les principales caractéristiques du microbiome cutané, dans la symbiose et dans la dysbiose, élucide les stratégies de rééquilibre du microbiote cutané et répond aux défis du développement de produits respectueux du microbiome par le biais d’études de l’interaction entre le microbiome cutané et les catégories substantielles d’ingrédients cosmétiques, tels que les surfactants, les composés lipophiles, les conservateurs, les parfums, les vitamines et les filtres UV. Les résultats présentés élucident la relation entre l’hôte, le microbiome cutané et l’utilisation de cosmétiques, qui pourrait servir d’outil pour le développement de cosmétiques respectueux du microbiome. Compte tenu de la popularité croissante de ce sujet, nous mettons également en évidence la nécessité de poursuivre les recherches axées sur la dynamique entre le microbiome cutané et les ingrédients cosmétiques.

## INTRODUCTION

The human skin operates as the body's primary defence against external hazards, providing physical, chemical, and immunological protection [1]. Covering an area of ~ 2 m^2^, it is distributed in microenvironments inhabited by various microorganisms that comprise the skin microbiota. In homeostasis, the skin microbiota benefits from lipids, salts, and proteins secreted by the skin, using them as a food source. In return, it aids in the process of integrity and repair of the host's skin barrier—a mutually beneficial relationship [2, 3, 4].

Skin microbiota plays a strong role in the immune system, where innate and adaptive immune responses for tissue repair and preservation are regulated by specific microbiota functions [1]. When the diversity and abundance of commensal microorganisms are unbalanced, the relationship with the host can become non‐symbiotic, resulting in dysbiosis. Aspects, such as infections, itching, redness, and dryness are associated with microbiota imbalance. In more severe cases, dysbiosis can trigger or worsen inflammatory dermatoses, such as acne vulgaris, atopic or seborrheic dermatitis, rosacea, and psoriasis [5, 6, 7].

Understanding the causes of dysbiosis is essential to accessing effective treatments. In this scenario, strategies for restoring diversity and relative abundance to rebalance the microbiome have been increasingly explored in the literature [8, 9, 10, 11, 12]. The approach that previously involved promoting asepsis in dysbiotic conditions is now focused on restoring the resident microbiota, responsible for various processes that maintain skin integrity and protect it against pathogens [13, 14].

The inclusion of key components, such as prebiotics, probiotics, and postbiotics, is essential for enhancing the diversity and abundance of commensal microorganisms, educating the immune system, and maintaining homeostatic skin condition [15, 16]. Prebiotics are nutrients, such as fibres and carbohydrates, that serve as energy sources for commensal microorganisms. Probiotics are viable cells of these commensal microorganisms, active or dormant, that can promote cosmetic benefits to the host at the application sites. Postbiotics, on the other hand, are represented by inanimate ingredients derived from probiotic fermentation with cosmetic benefits [15, 17, 18, 19].

There is a growing interest in topical products designed to support recovery or maintain skin microbiome balance, which has been noticed as a demand in the cosmetics industry for products compatible with the skin complex ecosystem. Nevertheless, several ingredients in cosmetic formulations can directly or indirectly influence this balance, such as harsh surfactants and non‐selective preservatives, widely present in daily skin care products. For modulation solutions with prebiotics, probiotics, or postbiotics, the non‐biotic ingredients present in topical formulations must demonstrate the lowest possible impact on the health of the skin microbiome or even improve its condition. This concept, known as microbiome‐friendly, illustrates a healthier relationship between cosmetics and the skin microbiome [13, 20, 21].

In addition to improving the balance of the skin microbiome, biotic compounds in microbiome‐friendly formulations can provide other cosmetic benefits. Sugars and specific extracts can offer anti‐ageing and antioxidant properties [22, 23], while probiotic cells (viable or lysate) and their products can present photoprotection, moisturization, and anti‐inflammatory properties [24, 25, 26]. Also, additional cosmetic benefits associated with prebiotics, probiotics, and postbiotics include anti‐acne, anti‐seborrheic, virucidal, deodorant, and anti‐pollution properties [10, 11, 27, 28].

This review aims to explore the concept of the skin microbiome and its relationship with the immune system in symbiotic and dysbiotic conditions, as well as to investigate recent strategies for rebalancing the skin microbiota and the challenges in developing microbiome‐friendly products based on non‐biotic ingredients and their interplay with the skin microbiota.

## SKIN MICROBIOME

Human skin hosts millions of microorganisms that coexist in a symbiotic relationship, where both benefit from this interaction [1]. Skin microbiota is considered the first line of defence against extrinsic factors, such as unknown opportunistic pathogens. The skin microbiota is vital for the metabolization of products excreted by epidermis layers and their appendages, in addition to preserving and stimulating the immune system, promoting skin homeostasis [3].

Recent advances in genomic sequencing techniques have enabled a greater understanding of the skin microbiome's diversity, especially regarding uncultivable microorganisms by traditional methods. Cultivation‐independent techniques, driven by advances in genomic technology, allow the identification and characterization of microorganisms through specific sequences such as 16S rRNA for bacteria, the most studied microorganisms, and 28S‐ITS for fungi. These modern approaches facilitate the detection of operational taxonomic units and the assessment of the relative abundance of each species [3, 5, 29].

The composition of the skin microbiota varies significantly between the different physiological areas of the skin, classified as moist, sebaceous, and dry. The distinct distribution of hair follicles, sweat glands, and sebaceous glands creates different conditions for the growth of microorganisms, resulting in a diverse ecosystem. In moist areas, such as the armpits, groin, and feet, *Staphylococcus* and *Corynebacterium* species predominate. In sebaceous skin areas, such as the face and chest, there is a greater abundance of lipophilic species, such as *Cutibacterium* (formerly *Propionibacterium*), which thrive in lipid‐rich, low‐oxygen environments. Conversely, in dry areas, such as the forearms and legs, *Proteobacteria* species are more abundant, reflecting the specific conditions of these locations [5, 6, 30].

Some specific microenvironments, such as sebaceous and sweat glands, are composed of a unique microbial community adapted to their particular conditions. In sebaceous glands, which are anaerobic and lipid‐rich environments, for example, *Cutibacterium* is predominantly colonized. The axillary region, which combines characteristics of moist and sebaceous areas, is primarily inhabited by Gram‐positive bacteria of the genera *Staphylococcus*, *Micrococcus*, *Corynebacterium*, and *Cutibacterium* [3].

In addition to the spatial distribution of microorganisms on the skin, endogenous and exogenous factors such as age, hormonal changes, lifestyle, genetic conditions, gender, and use of cosmetics and medications can also significantly influence the composition of the skin microbiota. Such changes in skin microbiota may be associated with various dermatological conditions, such as atopic dermatitis, acne, and psoriasis. This understanding has led to the development of therapies targeting skin microbiome as a new approach to treating skin diseases [6].

### Skin barrier function and skin microbiome

Lee & Kim [4] discussed the relationship between the skin barrier and the barrier created by the presence of the skin microbiome, in which the promotion of barrier protection occurs through a multifactorial process, interconnected by physical, chemical, and immunological impacts.

The physical impact is primarily related to the physiology of the epidermis, which is composed of different layers of stratified epithelial cells renewed through a process of cellular differentiation. This layered structure, combined with the abundant presence of structural proteins such as keratin and intercellular lipids, maintains a configuration of low permeability to pathogens. The epidermis also has a diverse population of resident immune cells, including Langerhans cells and T cells, which work together to constantly monitor the environment and coordinate appropriate immune responses, thus contributing to an integrated and effective defence system. Therefore, the physical barrier possesses mechanisms essential for the integrity of the skin tissue, in addition to harbouring a layer of commensal microorganisms that are also responsible for the skin's homeostatic state [2, 4, 30].

Regarding chemical impacts, barrier protection is related to metabolization by commensal microorganisms of products secreted by the skin. In some cases, products secreted by the glands are used as carbon sources by these microorganisms, such as urea present in sweat and lipids present in sebum. Lipophilic commensal microorganisms, such as *Cutibacterium* species, require essential nutrients produced by sebaceous glands, whose metabolite production contributes to skin barrier homeostasis [2]. Symbiotically, skin microbiota is essential in maintaining the acid mantle via organic acid metabolization, which creates an unfavourable environment for pathogen growth. Commensal microorganisms also positively contribute to stimulating the expression of antimicrobial peptides (AMPs) through the hydrolysis of fatty acids present in sebum. AMPs, in turn, actively participate in the body's immune response [31, 32].

In immunological impacts, the skin microbiota participates actively in innate and adaptive responses for tissue preservation, where the immune system is educated by the host microbiota for defence against external agents and repair [32]. *C. acnes* and *S. epidermidis*, for example, are responsible for controlling the expression of certain AMPs that are part of the immune response, in addition to inhibiting the growth of pathogenic microorganisms. In addition to the expression of AMPs, such as betadefencin and cathelicidins, which are present in greater abundance in the skin, resident microorganisms also act in the activation of T cells and IL‐1alpha, essential for the skin tissue repair process [5, 32, 33].

In particular scenarios, intrinsic and extrinsic factors negatively impacted the diversity and abundance of skin microbiota. As a result, changes in commensal populations and the presence of pathogens can affect the properties of skin tissue repair and skin homeostasis, triggering inflammatory conditions [31].

## IMPACTS OF DYSBIOSIS

The persistent imbalance between resident and transient microorganisms in the skin microbiota directly impacts its homeostatic state, leading to dysbiosis. This condition compromises the skin's barrier function, reducing its ability to retain water and protect against pathogens [4]. The skin microbiota plays an active role in maintaining hydration and skin barrier integrity. However, triggered by intrinsic and extrinsic factors, commensal microorganisms may cease to perform their symbiotic function, reducing protection against pathogens and compromising immune responses [34, 35]. As a result, the skin becomes more susceptible to infections, irritations, and exacerbated immune responses, which can cause or aggravate pre‐existing skin conditions. Dysbiosis is closely related to skin conditions such as acne, rosacea, dandruff, atopic dermatitis, and changes associated with ageing [4].

Clavaud et al. [29] compared the skin microbiota of healthy and dandruff‐affected individuals. The results revealed that the abundance of *S. epidermidis* and the fungus *Malassezia restricta* was higher in the dandruff group, while *C. acnes* was higher in the control group. These findings suggest that dysbiosis can alter the symbiotic relationship between commensal microorganisms and the host, impacting even species traditionally considered beneficial, such as S. epidermidis and *C. acnes*. Furthermore, the study highlighted the importance of balance between microorganisms from different kingdoms, demonstrating that homeostatic conditions are a multifactorial process.

*Acne vulgaris*, for example, is a dermatological condition that affects ~ 85% of adolescents. Located in the pilosebaceous units, a sebum‐rich microenvironment, it is a multifactorial process favoured by an imbalance in the microbiota residing in a specific niche. These sebaceous skin areas host lipophilic commensal microorganisms, which, under homeostatic conditions, metabolize the lipid content of sebum and transform it into fatty acids essential for skin health. However, with dysbiosis, there is an abrupt growth of *C. acnes* and a reduction in microbial diversity in the area, which favours inflammation, excessive sebum production, duct obstruction, and ultimately, the appearance of blackheads [31, 36].

Microbiota imbalance is also responsible for dryness, itching, and inflammation in other areas of the skin, which are conditions commonly observed in atopic dermatitis. Individuals with atopic dermatitis have a dysfunction in the expression of AMPs, such as beta‐defensins and cathelicidin, produced by commensal microorganisms such as *S. epidermidis*. Reduced AMP expression is associated with increased colonization by pathogenic microorganisms such as *S. aureus*, inducing allergic and inflammatory reactions, in addition to reducing skin barrier protection. Nakatsuji et al. [37] observed that *S. epidermidis* is more abundant in healthy individuals, while *S. aureus* is more prevalent in individuals with atopic dermatitis. In this case, recolonization of *S. epidermidis* is necessary for barrier repair, increasing the expression of AMPs, and consequently, the development of T cells that assist in the immune response.

Skin ageing presents physiological alterations that may contribute to microbiota imbalance. In this case, diversity and abundance changes of commensal and pathogenic microorganisms can lead to dysbiotic conditions. During ageing, skin presents a deficiency in the production of essential lipids, which consequently reduces the abundance of commensal microorganisms due to the low availability of nutrient sources, compromising the metabolic production of fatty acids. Therefore, reduction of skin hydration levels and tissue repair capacity is noticed in skin ageing, leading to dryness, loss of elasticity, and increased susceptibility to infections. Li et al. [38] found high susceptibility to colonization by more species in skin ageing, increasing the diversity of microorganisms that can accelerate skin ageing conditions, including *Cyanobacteria*, *Staphylococcus*, *Cutibacterium*, *Lactobacillus*, *Corynebacterium*, *Streptococcus*, *Neisseria*, *Candida*, and *Malassezia*.

### Cosmetic use and negative skin microbiome interactions

Cosmetic products are used daily by most of the world's population for cleansing, beautifying, and skin care [39]. There is a dual complex relation between the skin microbiome and cosmetics, where some cosmetic ingredients, such as lipids and waxes, can provide nutrients for commensal microorganisms, and on the other hand, some ingredients may be extrinsic factors associated with an imbalance in the skin microbiota, contributing to the onset or severity of disorders [40, 41].

Cosmetic ingredients, including those used daily, such as some classes of surfactants found in soaps, can alter the lipid and protein content of the stratum corneum and remain deposited on the skin for weeks [42]. Thus, the survival of commensal microorganisms is compromised by nutrient limitation, affecting the skin's homeostatic state [43, 44].

Rinse‐off cosmetics can also influence skin pH during application, as these products have a neutral or alkaline pH, while in its physiological condition, the skin's pH is 5.4–5.9 [45, 46, 47]. These variations can modify microbiome diversity, and consequently, impact immune response, hydration levels, and barrier protection function [32]. On the other hand, some products with a pH slightly lower than physiological (<5) were tested by Janssens‐Böcker et al. [41], where an increase in skin microbiome diversity was observed in both the control and treated groups, indicating that products with a pH lower than physiological pH pose less risk to skin microbiome diversity.

Some classes of preservatives also interact with the skin and remain deposited even after several baths, promoting prolonged chemical interactions and negatively impacting the skin microbiota [44, 48]. For example, the main commensal microorganisms present in the skin, *S. epidermis* and *C. acnes*, can be inhibited by a traditional preservative used in the cosmetic and pharmaceutical industries, methylparaben [33, 49]. At 0.5–1.5% methylparaben radically inhibits the population of these two commensal microorganisms within a few hours of contact, between 4 and 8 h [49]. On the other hand, despite the inhibitory and non‐selective effect of traditional preservatives, Murphy et al. [50] emphasize that the efficiency of these preservatives becomes more limited in real‐world use conditions, where the concentration of preservatives in cosmetic products can be diluted 5–10 times during application, causing less impact on the skin microbiome. Another factor highlighted by the work is the resilience and responsiveness of the skin microbiome to distinct external influences.

Understanding the interaction between chemical compounds present in cosmetics and the skin microbiota is essential for developing products that do not negatively impact the balance of the skin microbiota and host's health [42]. These findings reinforce the importance of strategies aimed at restoring the diversity and function of the skin microbiome, such as the use of ingredients that do not inhibit – and even promote – the diversity and abundance of commensal microorganisms for the treatment and prevention of dermatological conditions related to dysbiosis.

## STRATEGIES FOR REBALANCING SKIN MICROBIOME

Preventing and combating conditions that can lead to dysbiosis is essential for achieving homeostatic skin conditions. Advances in skin microbiome studies also provide more information about solutions focused on repairing the skin microbiota. The knowledge about the interactions of beneficial ingredients is increasingly deepened and applied to diverse scenarios, from the prevention or treatment of dysbiosis, as well as to clarify the connection between the skin and gut microbiomes, and emotions and mental health, known as the gut‐brain‐skin axis [51, 52].

In general, the strategy for achieving optimal skin functionality focuses on rebalancing cutaneous microbiota by normalizing the abundance and diversity of commensal microorganisms, in addition to controlling microorganisms or other pathogens, such as pollution, UV radiation, and specific chemical ingredients. These therapeutic strategies for skin microbiota rebalance can be accessed through prebiotics, probiotics, and postbiotics.

Prebiotics are nutrients added to topical products that selectively promote the growth of commensal microorganisms and the production of essential metabolites, such as short‐chain fatty acids (SCFAs). The production of SCFAs occurs through the fermentation of these compounds by commensal microorganisms. Prebiotics become key elements in modulating the skin microbiome, enhancing the immune response and presenting cosmetic benefits [18, 53, 54].

In topical products, probiotics are defined as viable cells of commensal microorganisms added to cosmetic products, which present essential functions in modulating the skin microbiome and promoting cosmetic benefits [17, 18]. Species of lactic acid bacteria or bifidobacteria are widely used as probiotics and have mechanisms that directly or indirectly contribute to the suppression of pathogens, producing compounds, such as AMPs, fatty acids, enzymes, vitamins, and exopolysaccharides [55]. Probiotics are also commonly used orally to restore the gut microbiota, but they can also be helpful to the skin microbiota through similar strategies. The gut‐skin‐brain axis can also induce positive systemic effects on the skin microbiota through therapies targeting the gut microbiome [53, 56].

Inanimate components derived from microbial origin can be classified as postbiotics or their related subclasses, such as parabiotics or paraprobiotics [17, 18]. Lysed probiotic cells, cell fractions, enzymes, peptides, SCFAs, and vitamins are examples of postbiotic compounds derived from bacterial and yeast fermentation [10, 19]. For topical applications, postbiotics are an excellent alternative to probiotics. Unlike probiotics, postbiotics do not comprise living cells, have an extended shelf life, are higher pH and temperature‐tolerant, and display similar modulating effects [51, 57].

Active ingredients, such as prebiotics, probiotics, and postbiotics, are applied in cosmetic products to maintain and prevent barrier function by modulating the skin microbiome. Probiotics, which consist of viable cells of beneficial bacteria, present several industrial challenges to be incorporated into cosmetic products, for example, (a) hostile environment provided for cosmetic semi‐solid products for probiotic cells surveillance; (b) contamination of different strains, including pathogens, affecting the safety and shelf life of product; (c) required specific storage conditions and use instructions; (d) regulatory issues gap, which probiotics probably exceed the current microbial limit in cosmetics, Bacteriological Analytical Manual (BAM) from Food and Drug Administration (FDA), for example, delimit microbial content of 1000 CFU/g for non‐eye‐area products [17, 18, 24, 51]. Regarding the challenges of incorporating probiotics into cosmetic products, Table 1 shows the diverse topical applications of prebiotics and postbiotics.

**TABLE 1:** Compounds with prebiotic and postbiotic potential for the skin microbiota.

Compounds	Potential treatment	Efficacy studies	Results	Claims	References
*Halomonas* levan polysaccharides and their modified forms	Prebiotic	Cell proliferation, wound healing, gene expressions of type I collagen, hyaluronic acid (HA), involucrin (INV), and filaggrin (FLG) in human keratinocyte (HaCaT), and fibroblast (PCs‐201‐012) cells	Induced gene expression of type I collagen, hyaluronan synthase 3, fillagrin and involucrin with the best fold changes, respectively: 2.63; 1.41; 1.74; and 6.43.	Anti‐ageing and indirect improvement of skin microbiota balance	[22]
Fructooligosaccharides (FOS)	Prebiotic	Growth inhibition/promotion of *S. aureus, S. epidermidis*, and SCFAs production	Cell proliferation of commensal microorganism strain, improvement of its SCFAs production by *S. epidermidis*, and reduction of *S. aureus* biofilm capability	Direct improvement of skin microbiota balance	[58]
Blackcurrant extract and its enzymatic form	Prebiotic	In vitro growth inhibition/promotion of *S. epidermidis, S. aureus*, and *C. acness*, and total polyphenol content	At a polyphenol concentration of 0.025 mg GAE/mL, both blackcurrant extract stimulates *S. epidermidis* while inhibiting *S. aureus* growth	Direct improvement of skin microbiota balance and antioxidant activity	[59]
Short‐chain fructooligosaccharides	Prebiotic	Bacterial strains competition on a reconstructed human epidermis in vitro model	At 0.5%, the prebiotic compound showed a promotion of the relative abundance of *S. epidermidis* and *C. acness*. Inhibition of *S. aureus* was noticed at 1.0%	Direct improvement of skin microbiota balance	[60]
2‐butyloctanol	Prebiotic	Clinical study analysis of axillary human microbiota by Metagenomic analysis (16S rRNA gene sequencing), using a deodorant roll‐on basis (placebo) with 3% 2‐butyloctanol applied once a day for 4 weeks (*n* = 12)	Change in relative abundance of *Corynebacterium* (odourcausing bacteria) compared with placebo or baseline	Direct improvement of skin microbiota balance and antimicrobial activity (deodorant)	[27]
Galactooligosaccharides (GOS)	Prebiotic	In vitro growth inhibition/promotion of *S. epidermidis* and *S. aureus* after application of hydrogel or gel emulsion with 5% of GOS	Promotion of *S. epidermidis* growth and inhibition of *S. aureus* in nutrient media, hydrogel, and gel emulsion	Direct improvement of skin microbiota balance	[23]
*Hylocereus undatus* extract	Prebiotic	Clinical study (*n* = 33) evaluating a topical gel‐cream containing 1% of the extract versus placebo, twice a day, for 28 days. Metagenomic analysis (16S rRNA gene sequencing), irritation, sensitivity, and TEWL (*n* = 26) analyses were conducted	Growth of microbiota diversity with the best result (22%) in the female group over 45 years and reduction of *Corynebacterium tuberculostearicum* abundance. Skin inflammation, redness, and TEWL were also reduced	Direct improvement of skin microbiota balance, anti‐ageing, and anti‐inflammatory activity	[61]
Polysaccharides extracted from *tremella*, dendrobium, and snow lotus (MP)	Prebiotic	Clinical study (*n* = 74) evaluating a topical propylene glycol aqueous solution with MP versus placebo, for 28 days. Metagenomic analysis was performed using 16S rRNA gene sequencing	Relative abundance of Firmicutes phylum (mainly *Staphylococcus* and *Bacillus*) increased from 20.9% to 82.1% (*p* < 0.05), while control solution did not show a significant difference	Direct improvement of skin microbiota balance	[9]
Enzymatic glucosylation of epigallocatechin gallate (EGCG‐G1)	Prebiotic	Evaluation of brightening properties using EGCG‐G1 cream, twice a day, with analysis at 0, 28, and 56 days in clinical studies in different skin types. In vitro growth inhibition/promotion of *Lactobacillus* sp. and *Bacillus* sp.	Promotion *L. acidophilus* growth at 27% using a 1% EGCG‐G1 concentration. Down‐regulation of melanogenesis pathways	Brightening properties and direct improvement of skin microbiota balance	[62]
*Polymnia sonchifolia* root juice (and) maltodextrin, and a fatty acid combination in solid lipid nanoparticles (SNL)	Prebiotic Postbiotic	Effect of holobiont tablet in virucidal activity (Vero cell), gene expression of interleukins 1, 6, 8, and 17, aquaporin‐3, antioxidant activity, collagen‐1 synthesis in human cells (monolayer and human equivalent skin) In vitro growth inhibition/promotion of *Lactobacillus* sp. and *Bacillus* sp.	Good virucidal activity in comparison to 70% alcohol, restoration of skin microbiota after use, maintenance or slight improvement of skin barrier protection biomarkers	Direct improvement of skin microbiota balance, virucidal activity, and barrier protection	[10]
Alpha glucan‐oligosaccharide and Pseudoalteromonas ferment extract (lysate)	Prebiotic Postbiotic	Clinical study (*n* = 25) of an anti‐ageing cream containing the mentioned prebiotic and postbiotic, applied twice a day, for 28 days, by metagenomic analysis (16S rRNA gene sequencing) at T0 and T28	βdiversity increase of bacteria at the species level compared with baseline (*p* < 0.001)	Direct improvement of skin microbiota balance	[63]
Inulin, butyloctanol, lactic acid, and pyruvic acid combination	Prebiotic Postbiotic	Clinical study with dry skin subjects (*n* = 53) applying a body wash and body lotion with or without pre/postbiotics for 6 weeks. Skin biophysical parameters, metagenomics (16S rRNA gene sequencing), and metabolomics were analysed at 0, 3, and 6 weeks	Higher bacterial sugar degradation in the prebiotic group, favouring diversity and abundance of commensal bacteria, as *Staphylococcus equorum*, *Streptococcus mitis*, and *Halomonas desiderata*. Skin hydration improvement was shown in both groups	Direct improvement of skin microbiota balance and skin hydration	[64]
*Lactococcus* Ferment Lysate	Postbiotic	SPF in vitro by UV spectroscopy	UVB spectrum absorption and SPF higher than homomenthyl salicylate, 4.75 ± 0.74 and 4.45 ± 0.45, respectively	UVB protection	[24]
Bacterial‐free compounds derived from *Latilactobacillus curvatus* fermentation	Postbiotic	In vitro HaCat cells and ex vivo investigation of postbiotic action in persistent intracellular *S. aureus* infections and wound healing	Postbiotic compounds showed good viability and reduction (*p* < 0.005) of *S. aureus* CFUs in the cell line. AMPs expression and anti‐inflammatory response (cytokine IL10) were found in the ex vivo model	Direct improvement of skin microbiota balance and skin barrier protection (wound healing)	[25]
Galactomyces ferment filtrate	Postbiotic	Transcriptomics analysis was performed using in vitro keratinocytes primary cells and ex vivo skin explants from female donors (*n* = 3)	TGM1 gene upregulation, related to cell differentiation, was shown in both models. CLDN4, CLDN1, SPRR2A and KRT13 gene upregulation was noticed in vitro	Improvement of skin barrier protection, anti‐inflammatory activity, and indirect improvement of skin microbiota balance	[26]
*Epidermidibacterium Keratini* ferment filtrate	Postbiotic	A prospective randomized split‐face clinical study on Asian women participants (*n* = 55) was performed for 3 weeks, analysing skin biophysical parameters and metagenomics (16S rRNA gene sequencing)	Significant increase (*p* < 0.001) of skin conductance, surface elasticity, dermal density, and TEWL reduction in comparison to baseline. The abundance of *Clostridium* and *Prevotella* was changed (*p* < 0.05)	Direct improvement of skin microbiota balance and anti‐ageing	[65]
Bacterial‐free compounds derived from *Bifidobacterium lactis* (HN019, B420, and Bi‐07 strains)	Postbiotic	In vitro growth inhibition/promotion of *M. furfur* and *C. acnes*s analysed by optical density (OD = 600 nm)	After 48 h, *Bifidobacterium lactis* presented an inhibition rate of 90% of microorganisms related to acne vulgaris and seborrheic dermatitis	Direct improvement of skin microbiota balance, anti‐acne, and anti‐seborrheic	[11]
*Lactobacillus plantarum* ferment lysate	Postbiotic	Pilot clinical study applying an anti‐acne lotion in subjects with mild‐to‐moderate acne (*n* = 22) over 4 weeks. Skin biophysical analysis was conducted	TEWL and sebum production were significantly decreased (*p* < 0.05) compared with baseline.	Anti‐acne and indirect improvement of skin microbiota balance	[8]
*Bacillus coagulans* (MTCC 5856) ferment filtrate extract	Postbiotic	Clinical randomized, open‐label, comparative study with mild‐to‐moderate acne subjects (*n* = 64) for 3 weeks. Skin biophysical analysis was performed to evaluate the efficacy of postbiotic and benzoyl peroxide treatment	Antimicrobial activity at acidic pH conditions. Postbiotic treatment showed a comparable efficacy to standard treatment; the highest efficacy results were found in closed comedones	Anti‐acne and indirect improvement of skin microbiota balance	[66]
Kiwi‐derived yeast extract	Postbiotic	Ex vivo and clinical, randomized, and triple‐blind study for 8 weeks to evaluate three cosmetic formulas. Skin biophysical (*n* = 110), metagenomics (*n* = 97) for bacterial and fungal gene sequencing (16S rRNA and 1TS1), and gene expression (ex vivo) analysis were conducted	All cosmetic formulas improved skin hydration parameters, even after environmental aggressors; no significant difference was found. Capalase‐14, collagen‐1, and elastin gene expression was increased (*p* < 0.005) in the postbiotic content formula. Also, the bacterial diversity of *S. epidermidis* and *Ralstonia*, which metabolizes pollutants (ex, PAHs), was enriched	Direct improvement of skin microbiota balance, anti‐pollution, anti‐ageing	[28]

The topical use of prebiotics and postbiotics promotes the balance of the skin microbiome, improving environmental conditions for beneficial strains to grow selectively, according to in vitro, in vivo, and ex vivo assays. Several compounds in the table demonstrate the selective growth of commensal microorganisms, such as *S. epidermidis* and *Lactobacillus* sp., in addition to the inhibition of microorganisms with potential pathogens, such as *S. aureus* and *M. furfur*, indicating that the proposed treatments modulated the skin microbiome. These active compounds also offer other functional benefits, with physiological properties such as barrier repair, reduction of TEWL, expression of genes related to hydration and elasticity (collagen‐1, filaggrin, aquaporin, CLDN‐1, hyaluronic acid), anti‐ageing, anti‐acne, anti‐pollution, and photoprotection. These benefits, in turn, may be directly or indirectly related to the increased diversity and abundance of commensal microorganisms.

## CHALLENGES AND NEW PERSPECTIVES OF COMMON COSMETIC INGREDIENTS

Deepening the dynamics of the skin microbiome and its modulation offers new solutions to standard treatments for acne vulgaris, seborrheic dermatitis, and premature ageing [11, 31, 67]. However, when adopting the microbiome‐friendly concept, non‐biotic ingredients (not classified as prebiotics, probiotics, or postbiotics) present in cosmetic formulations should not negatively alter the skin microbiome [21].

The challenge of developing microbiome‐friendly cosmetics involves selecting commonly used ingredients with different chemical compositions. Cosmetic ingredients in lotions, creams, and soaps can chemically alter skin microenvironments, remaining deposited for 0.5–1.9 weeks after use, causing changes in the abundance and diversity of resident microorganisms [42, 68]. Selecting ingredients that have a low impact on the balance of the skin microbiome, such as mild surfactants and emulsifiers, and selective preservative systems, can positively contribute to the microbiome‐friendly concept.

Numerous ingredients are recognized for their potential to interfere with the skin microbiome by altering its lipid composition, pH, conductance, or photoprotection. However, the crucial question is *how* this happens. This section presents some classes of ingredients commonly used in cosmetics and their different interactions with the skin microbiome.

### Surfactants and emulsifiers

This class includes essential ingredients for the preparation of a semi‐solid product, such as amphiphilic molecules and polymers, essential for creams, lotions, cleansers, and gels.

In some cases, harsh surfactants and alcohols negatively interact with the skin microbiome through drastic changes in the skin's lipid mantle [69]. Anionic surfactants such as SLS and SLES are commonly used in rinse‐off preparations and are associated with potential irritant effects. Leoty‐Okombi et al. [70] found that the use of 0.5% SLS in healthy women in a patch condition for 24 hours can reduce the levels of barrier protection and beneficial microbial communities on the skin, increasing the risk of dysbiosis.

For Lokhande et al., [13] microbiome‐friendly cosmetic formulations should replace harsh surfactants with gentler alternatives, such as decyl glucoside and sodium cocoyl glutamate, bio‐based mild surfactants capable of removing impurities from the skin without drastically affecting its lipid mantle, and consequently, the balance of the skin microbiome. On the other hand, Zhao et al., [71] observed that the use of sodium lauroyl sarcosinate, another bio‐based mild surfactant present in several cosmetic preparations, can be aggressive to the skin microbiota. The application of a soap with 8% surfactant for 3 weeks altered the chemical composition of the skin barrier by increasing phosphatidylglycerol and phosphatidylcholine and reducing ceramides, leading to a reduction in alpha diversity and facilitating the colonization of pathogens more resistant to changes in the skin ecosystem.

Cationic surfactants and polymers are commonly used in hair care products and also present antimicrobial properties; however, they may possess a high irritancy potential for the skin, depending on their structure [72, 73]. Due to their antimicrobial activity, it is believed that cationic surfactants and polymers can also negatively affect the balance of the skin microbiota. Interestingly, Machado et al., [10] demonstrated that the application of cetyltrimethylammonium chloride in a non‐alcoholic hand sanitizer, as a base component for obtaining a nanostructured system, presented a lower risk to the skin microbiome compared with 70% alcohol. The authors also demonstrated that the balance of the skin microbiota was restructured 2 h after application of the non‐alcoholic hand sanitizer, making it a healthier alternative to the skin microbiome than 70% alcohol.

The drastic alteration in the chemical composition of skin caused by surfactants can lead to imbalanced skin microbiota and trigger adverse reactions. Ananthapadmanabhan et al. [43] consider that amphoteric or nonionic compounds have less irritation potential than anionic and cationic compounds. Replacing or reducing mild surfactants with less irritating molecules can reduce the impact on the skin's microbiome and promote gentler cleansing.

In the case of emulsifiers, which are indispensable ingredients in creams and lotions, many are nonionic, such as fatty alcohols, glycerol derivatives, and fatty acids. These latter are vital components for the maintenance of the skin's lipid mantle (e.g., saturated fatty acids like stearic, palmitic, oleic, and myristic acids) and can be incorporated into cosmetic emulsions due to their emulsifying and, in some cases, emollient properties [74].

Emulsifiers derived from glycerol and fatty acids can be an alternative for microbiome‐friendly formulations, such as polyglyceryl‐based emulsifiers. This class of emulsifiers is obtained from renewable sources and provides stability to the dispersion between immiscible phases, possessing emollient properties. Schulte et al. [75] investigated the interaction between the emulsifiers polyglyceryl‐10 oleate and polyglyceryl‐10 stearate with the skin microbiota. Neither emulsifier showed significant changes in alpha diversity after 4 weeks of topical use (*n* = 24) of a formulation containing 5% (w/w) of each emulsifier.

Furthermore, evidence suggests that Tween 80, a nonionic emulsifier derived from oleic acid, traditionally used in pharmaceutical and cosmetic preparations, does not negatively interact with the skin microbiome and even functions as a pathogen inhibitor [76]. However, the interaction between chemical compounds and the skin microbiome can present specific complexities, as is the case with Tween 80. The quantity, nutrient availability, and hydrophobicity of microorganisms can determine whether this relationship is beneficial or not. Although it inhibits a pathogenic microorganism from a low concentration of 0.1%, according to Nielsen et al. [77], Tween 80 can also affect the growth of commensal microorganisms at higher concentrations, as seen by Menberu et al. [76], when demonstrating the inhibitory effect at a concentration of 1% (w/w) against *S. epidermidis* in a medium with little nutrients.

Amphiphilic compounds are essential for the development of cleansers, conditioners, creams, and lotions. Some compounds traditionally used in the cosmetics industry can have adverse reactions, including skin microbiome, such as harsh surfactants. Therefore, the necessity to reduce or replace these compounds is increasingly emerging for the development of cosmetics to interact positively with the skin ecosystem as a whole [78].

### Lipophilic compounds

Lipid components and fatty acid esters used in cosmetic products can influence the behaviour of bacterial and yeast species of the skin microbiome [69]. Dobler et al. [40] observed that most *Malassezia* species are capable of “feeding” on lipid compounds, such as avocado and olive oils, carnauba and beeswax, and fatty acids with more than 12 carbons. Furthermore, esters such as isopropyl palmitate and capric/caprylic triglycerides underwent hydrolysis and were transformed into fatty acids and alcohols through the capacity of microorganisms in metabolizing lipids. *Malassezia* spp. are yeasts that predominantly occupy sebaceous areas and depend on the host's lipid source for growth. The metabolism of ingredients commonly used in cosmetics for the growth of a yeast species may be of great interest in the development of microbiome‐friendly products.

The use of ceramides in products designed to promote hydration and/or maintain the skin barrier is strongly recommended, as ceramides have lipid matrix repair and barrier protection properties [4, 78]. In cases of atopic dermatitis or intense skin dryness (xerosis), replenishing ceramides in the stratum corneum is essential [79, 80]. Combined with other components present in the skin, such as free fatty acids and cholesterol, ceramides help reduce TEWL and the penetration of aggressive agents, favouring the homeostatic condition of the skin microbiome [81].

Emollient ingredients have positive interactions with the skin microbiome and are recommended for moisturizing formulations, even in conditions of dysbiosis [82]. Glatz et al. [83], for example, demonstrated that treatment with a topical preparation containing emollients can help acidify the skin and increase microbial diversity in atopic dermatitis.

### Antimicrobial compounds

As previously described (see section “Cosmetic Use and Negative Skin Microbiome Interactions”), several antimicrobial compounds used as preservative systems in cosmetic products exhibit broad‐spectrum antimicrobial activity, which drastically affects the population of commensal microorganisms. Some combinations of glycols, antioxidants, and chelators exhibit selective antimicrobial activity against pathogens. Pinto et al. [48] described the combination of hydroxyacetophenone, phenylpropanol, propanediol, caprylyl glycol, tocopherol, and tetrasodium glutamate diacetate as effective against *S. aureus* and tolerable to the main commensal skin microorganisms, *S. epidermidis* and *C. acnes*. In addition to products where homeostatic conditions must be restored, the authors suggested the combination of phenylpropanol, propanediol, caprylyl glycol, tocopherol, and disodium EDTA.

Shao et al. [84] found low inhibitory activity for *S. epidermidis* compared with *S. aureus* at low concentrations of 1,2‐hexanediol. While alkanediols, such as 1,2‐butanediol and 1,2‐pentanediol, promoted *S. epidermidis* growth at low concentrations, indicating that these components can be applied within the microbiome‐friendly concept. Furthermore, the authors highlight that the concentration of these compounds may considerably influence their interplay with the skin microbiome. Phenoxyethanol, for example, characterized as a gentle preservative, presented a minimum inhibitory concentration of 0.5% for *S. epidermidis* and *C. acnes*.

### UV protection compounds

Exposure to UV radiation, combined with additional extrinsic factors, affects skin integrity, and consequently, the balance of its microbiome. Under conditions of dysbiosis, UVR can drastically affect skin condition, primarily due to an imbalanced skin microbiome [85]. However, Patra et al. [86] demonstrated that the skin microbiome, despite being affected by UVR, can also exert a positive effect on photoprotection, where the skin microbiome acts by modulating gene expression to reduce immunosuppressive responses triggered by UV radiation.

The skin microbiota is exposed to UV radiation in the same way as its host, and its defence mechanisms against radiation remain poorly studied [85, 87]. Understanding the dynamics between UVR and the skin microbiome– as well as recurring photoprotection practices– can favour the development of personalized photoprotective products.

Schuetz et al. [87] compared an SPF 20 sunscreen containing ethylhexyl salicylate, octocrylene, bis‐ethylhexyloxyphenol methoxyphenyl triazine, and butyl methoxydibenzoylmethane as UV filters with a placebo formulation by simulating solar radiation in female volunteers (*n* = 10). As a result, relative diversity was primarily influenced by interindividual factors rather than the proposed treatments, with no significant changes between the placebo and the sunscreen. The absence of negative interactions between microbial diversity and sunscreen may be an indication that the organic UV filters present in the composition do not affect skin homeostasis.

Arantes et al. [88] investigated the impact of organic UV filters on the abundance and diversity of the main commensal microorganisms. Due to the high lipophilic content in sunscreen formulations, the hypothesis was that prolonged use of sunscreen can lead to inflammatory conditions and increased oiliness. In the study, a gel‐based cosmetic formulation containing ethylhexyl methoxycinnamate, ethylhexyl salicylate, methyl anthranilate, and octocrylene was applied twice daily for 10 days by a group of volunteers (*n* = 6) of men aged 19–23 years without acne, with the pure gel base serving as a control. Analysis of the material collected after treatment (16S ribosomal rRNA gene) showed no difference in the abundance and diversity of species such as *C. acnes* and *S. epidermidis* between the groups. Although the study population was small, no evidence was found that organic UV filters can lead to dysbiosis. Furthermore, the incorporation of organic UV filters in a gel base may also have contributed to the treatment's success, reflected in microbial taxonomic levels.

Inorganic filters, such as TiO_2_, are widely used in sunscreens, in different sizes, coats, and charges. In the study by Rowenczyk et al. [89], coated TiO_2_ nanoparticles (TNPs) with different charges were evaluated. Polar TNPs impacted the microbial growth kinetics of hydrophilic species such as *P. fluorescens* and *S. aureus* in emulsions stored at 40°C for 50 days, while more nonpolar TNPs had less impact on microbial growth. *P. fluorescens* and *S. aureus* species can be considered contaminants in cosmetics and potential pathogens in the skin microbiome under imbalanced conditions. Notably, coated TiO_2_ is frequently incorporated in sunscreen formulations and is deposited on the skin surface, contributing to a direct interaction with the skin microbiome. Thus, understanding the impact of TNPs in their multiple configurations (size, charge, polarity) in cosmetic formulations can help understand the relationship with the skin microbiome.

### Vitamins

Vitamins are widely used as cosmetic active ingredients for their antioxidant, anti‐inflammatory, and barrier protection properties.

Vitamin A and its derivatives regulate the immune system through pro‐inflammatory responses against pathogens via the expression of Toll‐like receptors 2 and 3 and AMPs. The effect of vitamin A on AMP expression also impacts the skin microbiome, where there is evidence of anti‐acne properties by reducing the abundance of *C. acnes* and antifungal properties by inhibiting *C. albicans*, *Aspergillus* spp., and *Microsporum* spp. In cases of atopic dermatitis and psoriasis, vitamin A may also have beneficial effects in controlling *S. aureus* [90, 91, 92].

Although there are few studies on their interactions with the skin microbiome, vitamins C and E are powerful allies in combating oxidative stress and modulating inflammation. Vitamin C contributes to skin homeostasis, which may be related to its collagen synthesis, ceramide synthesis, and free radical inhibition. Vitamin E, on the other hand, plays a key role in the first line of defence against cellular lipid peroxidation and also in protecting the skin's lipid composition, indirectly promoting the diversity of lipophilic microorganisms [91, 93].

Vitamin B5 presents barrier protection and hydration maintenance properties, being a key component in many cosmetic products. Qaizar et al. [94] evaluated the interaction of the skin microbiome in a representative real‐world condition with a product containing D‐panthenol for 4 weeks in a population of different ages and sexes. As a result, the diversity and abundance of the skin microbiome were not affected using D‐panthenol in the different groups. The study by Stettler et al. [95] also demonstrated that the use of D‐panthenol does not negatively impact the bacterial community on the skin.

### Fragrances

Although present in many cosmetic products, fragrances of natural or synthetic origin also interact with the skin microbiome in a way that remains unclear. Fragrances contain volatile components that interact with the skin microbiome, particularly in sebaceous areas where *C. acnes* is predominant. Fragrances derived from essential oils, rich in terpenes, may exhibit antimicrobial activity, but studies regarding their interaction with commensal microorganisms are lacking [96].

The interactions between volatile organic compounds (VOCs) and the skin microbiome have been increasingly studied, where the skin microbiome plays a key role in releasing VOCs, carrying small chains of volatile fatty acids [97]. Through the metabolization of sebum and sweat, bacteria such *as Staphylococcus*, *Corynebacterium*, and *Cutibacterium* can produce VOCs, leading to alterations in body odour as a distinct individual manifestation and probably influencing the olfactory characteristics of fragrances.

The relationship between cosmetic products and the skin microbiome is considered highly complex, with millions of microorganisms interacting with various chemical compounds in different topographical regions of the skin [40]. Modulating the skin microbiome has been used as an alternative to traditional treatments for dermatoses, as well as for maintaining skin health in beauty cosmetics [54, 98]. Therefore, there is a growing need for new technologies to develop beneficial products for skin microbiomes. Non‐biotic ingredients used in cosmetic formulations can interact with the skin microbiome in different ways, depending on their chemical structure, concentration, application site, and host condition.

## CONCLUSION

The advance of sequencing techniques has brought novel insights into a broad spectrum of areas, including the skin microbiome. The delicate relationship between the host and its resident microorganisms highlights the critical demand for new topical products focused on balancing or not affecting the skin microbiome. The cosmetic industry is experiencing a significant transition aimed at developing products that enhance or preserve skin microbiota health. The use of prebiotics, probiotics, and postbiotics is recommended for conditions, such as dryness, premature ageing, and inflammatory dermatoses. However, incorporating probiotics into cosmetic bases is still considered a challenge for the product's antimicrobial protection and the viability of probiotic microorganisms. Applying the microbiome‐friendly concept offers new perspectives for a healthy relationship between cosmetic use and skin microbiome. The interaction of non‐biotic cosmetic ingredients with the skin ecosystem is a highly relevant topic for the development of microbiome‐friendly cosmetics. Also, a mixture of cosmetic ingredients, whether biotic or non‐biotic, in a final formulation could lead to different interactions, emphasizing the complexity of understanding the dynamics between cosmetics and the skin microbiome. However, it is still an emerging area of research that requires increased visibility; for this reason, we strongly recommend conducting more studies to clarify the dynamics between the skin microbiome and the different classes of cosmetic ingredients.

## AUTHOR CONTRIBUTIONS

Conceptualization: Vânia Leite‐Silva and Patricia Lopes; Formal analysis: Yasmin Rosa Santos; Funding acquisition: Vânia Leite‐Silva; Investigation: Yasmin Santos; Project administration: Vânia Leite‐Silva; Supervision: Patricia Lopes and Vânia Leite‐Silva; Writing – original draft: Yasmin Santos; Writing – review & editing: Vânia Leite‐Silva, Newton Andreo‐Filho and Patricia Lopes. All authors have read and agreed to the published version of the manuscript.

## FUNDING INFORMATION

This research received no external funding.

## CONFLICT OF INTEREST STATEMENT

The authors declare no conflicts of interest.

## Data Availability

The data that support the findings of this study are available from the corresponding author upon reasonable request.
